# Did industrial and export complexity drive regional economic growth in Brazil?

**DOI:** 10.1371/journal.pone.0313945

**Published:** 2024-12-05

**Authors:** Ben-Hur Francisco Cardoso, Eva Yamila da Silva Catela, Guilherme Viegas, Flávio L. Pinheiro, Dominik Hartmann

**Affiliations:** 1 Departamento de Economia e Relações Internacionais, Universidade Federal de Santa Catarina, Florianópolis, SC, Brazil; 2 NOVA Information Management School (NOVA IMS), Universidade Nova de Lisboa, Lisbon, Portugal; Visva-Bharati University: Visva-Bharati, INDIA

## Abstract

Research on productive structures has shown that economic complexity conditions economic growth. However, little is known about which type of complexity, e.g., export or industrial complexity, matters more for regional economic growth in a large emerging country like Brazil. Brazil exports natural resources and agricultural goods, but a large share of the employment derives from services, non-tradables, and within-country manufacturing trade. Here, we use a large dataset on Brazil’s formal labor market, including approximately 100 million workers and 581 industries, to reveal the patterns of export complexity, industrial complexity, and economic growth of 558 micro-regions between 2003 and 2019. Our results show that export complexity is more evenly spread than industrial complexity. Only a few—mainly developed urban places—have comparative advantages in sophisticated services. Regressions show that a region’s industrial complexity is a significant predictor for 3-year growth prospects, but export complexity is not. Moreover, economic complexity in neighboring regions is significantly associated with economic growth. The results show export complexity does not appropriately depict Brazil’s knowledge base and growth opportunities. Instead, promoting the sophistication of the heterogeneous regional industrial structures and development spillovers is a key to growth. This study demonstrates that industrial complexity, which accounts for all employment sectors, provides a more accurate basis for designing effective and inclusive industrial policies in emerging economies like Brazil, compared to export-based complexity.

## Introduction

Geographical differences in productivity and growth prospects can be largely explained by differences in the set of activities carried out at each location. In other words, what a region produces matters to achieve certain levels of income and well-being. Based on this idea, several measures of Economic Complexity—such as the ECI [1], the Fitness index [2, 3], and GENEPY [4]—have been proposed to characterize productive structures. These measures—making use of dimensionality reduction techniques that capture the variety and ubiquity of an economy’s productive output—are important determinants of economic growth [5], socioeconomic development [6] and environmental sustainability [7–11]. The key underlying rationale is that it the expected economic return and level of value-added knowledge depends upon whether an economic agent (a country, region, firm, or worker) specializes in economic activities that many or few other agents can do.

Economic complexity literature has traditionally used international trade data at the country level to estimate country and product-level complexity indicators [1, 2, 12]. However, it seems that export-based complexity measures are inadequate in comprehensively capturing knowledge, productive capabilities [13], and self-discovery processes [14] in all economic activities, such as non-tradeable services [15], and present significant biases when studying regional dynamics in continental-sized countries such as Brazil or the USA. Indeed, the data entry of exports might not necessarily be the actual production place of a good but instead the registry location of exports [16]. In recent years, researchers have turned their focus to patents [17–20], academic output [21–23], workers’ skills [24], and industry [20, 25–29] data to estimate the complexity of economies. Like exports, these datasets capture the intensity of an activity (e.g., exports, patents, academic production, industries, etc.) at different regional units (e.g., cities, municipalities, regions, or countries) to estimate the complexities of activities and regions. Hence, given the multiple ways one can estimate regional complexity, it is natural to ask which is the most appropriate measure of economic complexity.

Research on regional complexity in developed economies (e.g., Europe and North America) tends to focus on patents to study technology complexity [17, 18, 20], which seems natural as many of these countries can be considered at the technology frontier [30] and compete for adoption or leadership in new technological domains. However, the same cannot be said to the same extent for regions in emerging economies, where patent production is regionally sparse. Hence, a substantial share of development research on Brazil and Latin America still focuses on trade data [31–35]. However, it makes a difference if the policy focus is on protecting and promoting export sectors (e.g., via tariffs or better international port infrastructure) or whether the focus is on issues such as knowledge-based business sectors or within-country manufacturing trade (e.g., via better ICT infrastructure, roads, service spaces).

While recent works have investigated economic complexity based on industrial and occupational data [16, 20, 26–28, 36–39], little emphasis has been put on exploring what is an adequate measure of economic complexity to depict regional development dynamics in developing and emerging economies, and how different measures adequately explain regional economic growth.

Here, we estimate two measures of regional economic complexity across 558 micro-regions of Brazil using industry (IndECI) and export (ECI) data between 2003 and 2019. We then compare these Economic Complexity measures, showing that while the former shows a temporal tendency to increase agglomeration (i.e., build-up of spatial correlations), the latter does not. Still, they capture several stylized facts from economic complexity literature. Finally, we show that industrial complexity is more relevant than export complexity for regional economic growth. Nonetheless, regions also positively benefit from their neighboring regions’ industrial and export complexity levels, arguably due to positive economic spillovers of demand, infrastructure, and knowledge.

In Brazil, the inward-looking and unequal development of the industrial sector can be traced through significant events in its recent economic history. Brazil, a vast country with a large domestic market as early as the 1950s, facilitated industrialization in the 1930s following the coffee economy crisis. Industrial policies initiated during the Vargas government and intensified with the Target Plan under Kubitschek further propelled this process. By the 1970s, the military regime’s National Development Plans led to substantial industrial growth [40]. Until the 1980s, Brazil experienced productivity gains, economic growth, and financial integration, making it South America’s most complex and diversified economy. However, this industrial growth followed an inward-looking model centered on import substitution. The 1980s saw the end of external financing and a debt crisis, while the 1990s’ reintegration with global markets failed to reignite economic growth, resulting in stagnation. In the 2000s, new industrial policies were introduced [41], but these efforts could not prevent the concentration of dynamic activities in localized areas. Some activities positively impacted aggregate productivity, while others showed negative growth [42].

While traditional structuralist economics have pointed to the external trade dependence of Brazil and other Latin American economies during outward-looking production systems in colonial and post-colonial times [43–46], recent decades have seen a shift. Different entrepreneurial activities and inward-looking policies have contributed to the diversification of local and national services as well as local manufacturing activities in Brazil. This aligns with the general trend of the increasing importance of services in the world economy, including finance, administration, software, digital and servitized products [15, 47, 48]. Consequently, export data alone does not provide a comprehensive picture of the skills, productive capabilities, and diversification opportunities of economies.

Notably, our study shows that, due to many regions exporting natural resource-based products, export complexity is more evenly distributed across Brazil compared to the higher concentration of industrial complexity. This does not mean that an overall lack of export complexity in Brazil is not a bottleneck for its economic development and growth. However, the differential in terms of knowledge and income creation across microregions appears to be increasingly found in non-tradables, services, servitized products, or specialized goods for the large internal market rather than in tradable goods such as natural resource-based products alone, at least in the case of Brazil between 2003 and 2019.

## Materials and methods

We use international trade data from the *Growth Lab of Harvard University* [49]. The dataset covers trade between 242 countries between 2003 and 2019 in 1260 traded products classified according to the Harmonized System (HS) at the 4-digit level in the 1992 version. From COMEX, we sourced the FOB export value of each product by Brazilian municipality and month [50]. We aggregated the data to micro-regional [51] and annual levels. From the *Relação Anual de Informações Sociais* (RAIS), we make use of data that informs on the number of hours worked annually in each industry and municipality in Brazil between 2003 and 2019 within the formal labor market. The 581 industries are encoded using 5-digit version 2.0 of the *Classificação Nacional de Atividades Econômica* (CNAE2.0). The data is aggregated at the micro-region level [51].

We did not explore the effects of patents and academic complexity for qualitative reasons. Patent applications are comparatively scarce [52] and thus seem inadequate for most regions and reduce our data considerably, apart from known problems of patents often being a measure of knowledge appropriation skills (and with a large share of foreign company applicants). Academic research output should be explored in more detail in the future. However, recent work on multidimensional economic complexity [23] points to a lower predictive power of research complexity than other ECI data sources. Moreover, it is known that research output in Brazil is mainly driven by public federal universities whose locations follow, to a significant extent, the population distribution as well as political reasons for broader access to higher education, and thus may also not depict the de-facto differences in productive capabilities well.

Finally, we measure the micro-regional real GDP per capita (GDPpc) and micro-regional number of inhabitants (population) using data from *Instituto Brasileiro de Geografia e Estatística* (IBGE) at the municipality level [53] that was aggregated at the micro-regional level [51]. We deflate the GDP to a 2010 base using the Brazilian Consumer Price Index [54]. Unfortunately, there is no data on other attractive micro-regional controls, such as unemployment and the share of the population living in an urban area. While we have noted the lack of detailed regional controls in our discussion, it is essential to clarify that our analysis includes regional fixed effects. These fixed effects partially account for regional differences and provide a more nuanced understanding of the relationships examined. Subsequently, we present our export- and industrial-based complexity measures in line with past works [1, 55]. It is noteworthy, though, is that the micro-regional level provides an analysis level due to conceptual reasons (designed by the statistical institute to capture connected labor markets).

### Exports and industry complexity

Let $X_{r,i}^{t}$ measure the revealed intensity of an activity *i* in region *r* on year *t*. We defined the Revealed Comparative Advantage (RCA) of region *r* in activity *i* as

$$\begin{array}{c} {\text{RCA}}_{r,i}^{t}=\frac{X_{r,i}^{t}}{\sum_{j}X_{r,j}^{t}}\frac{1}{Z_{i}^{t}} \end{array}$$ (1)

the upper term measures the relative intensity of activity *i* in the region *r*, and $Z_{i}^{t}$ provides a baseline expectation of the relative intensity of such activity from a typical region. This is equivalent to the Location Quotient and the Balassa Index [56].

We estimate the RCA using both industry and export intensity. In that sense, for industries, we measured intensity through the number of hours worked in the micro-region, and the baseline corresponds to the share of the number of hours spent in an industry *i* across the entire country, that is,

$$\begin{array}{c} Z_{i}^{t}=\frac{\sum_{r^{\prime }}X_{r^{\prime },i}^{t}}{\sum_{r^{\prime },i^{\prime }}X_{r^{\prime },i^{\prime }}^{t}} \end{array}$$ (2)

For exports, we measure intensity through the total reported exports in USD of a region in a given product, and, following previous works [57, 58], we consider the product’s share in global international trade between countries as a baseline ($Z_{i}^{t}$).

An RCA greater/lower than one implies that a region reveals an intensity on a given activity more/less than what we expect from a typical region. Next, we can represent the regional division of activities through a specialization matrix *M*^*t*^, with entries $M_{r,i}^{t}$ equal to 1 if the micro-region *r* ∈ *R* reveals a significant intensity of activity *i* in the year *t* (${\text{RCA}}_{r,i}^{t}>1$) and 0 otherwise.

Following standard methods from economic complexity [58], the complexity of a micro-region is the average complexity of the activities in which it has specialized in. As such, we can use the Product Complexity Index (PCI) estimated from international trade data [58] to obtain the complexities of each exported product and use the average to estimate each region’s export Economic Complexity Index (ECI):

$$\begin{array}{c} {\text{ECI}}_{r}^{t}=\frac{1}{M_{r,*}^{t}}\sum_{i}M_{r,i}^{t}{\text{PCI}}_{i}^{t} \end{array}$$ (3)

The intuition behind ECI is that sophisticated economies are diverse and export sophisticated products (with a high PCI) which produced by only a few diverse countries. ECI uses this variation in countries’ diversity and products’ complexity to measure a region’s productive structure, reflecting the sophistication of its products. However, in a developing country like Brazil, the export-competitive sectors are not necessarily the most sophisticated ones. In such cases, important sophisticated sectors compete with imports but are not sufficiently competitive to be exporters. Therefore, a better way to capture the sophistication of an economy that is not export-driven is to consider all sectors, not just the export-oriented ones, which in Brazil are primarily in the primary sector. To do so, we use the industrial employment data. However, we need to estimate both quantities from the existing industry data. As such, consider,

$$\begin{array}{c} K_{r}^{t}=\frac{1}{M_{r,*}^{t}}\sum_{i}M_{r,i}^{t}Q_{i}^{t} \end{array}$$ (4)

as the complexity of a micro-region ($K_{r}^{t}$) given the complexities of industries ($Q_{i}^{t}$). Conversely, the complexity of activity ($Q_{i}^{t}$) is the average of the complexity of the micro-regions ($K_{r}^{t}$) specialized in it:

$$\begin{array}{c} Q_{i}^{t}=\frac{1}{M_{*,i}^{t}}\sum_{r}M_{r,i}^{t}K_{r}^{t} \end{array}$$ (5)

which, after some manipulations, leads to:

$$\begin{array}{c} K_{r}^{t}=\sum_{r^{\prime }}{\hat{M}}_{r,r^{\prime }}^{t}K_{r^{\prime }}^{t} \end{array}$$ (6a)

$$\begin{array}{c} {\hat{M}}_{r,r^{\prime }}=\sum_{i}\frac{M_{r,i}^{t}M_{r^{\prime },i}^{t}}{M_{r,*}^{t}M_{*,i}^{t}} \end{array}$$ (6b)

and

$$\begin{array}{c} Q_{i}^{t}=\sum_{j}{\tilde{M}}_{i,j}^{t}Q_{j}^{t} \end{array}$$ (7a)

$$\begin{array}{c} {\tilde{M}}_{i,j}=\sum_{i}\frac{M_{r,i}^{t}M_{r,j}^{t}}{M_{r,*}^{t}M_{*,i}^{t}} \end{array}$$ (7b)

To solve these recursive equations, it is enough to identify that the complexity vector of the regions *K*^*t*^ is an eigenvector of ${\hat{M}}_{t}$ and the complexity vector of the activities *Q*^*t*^ is an eigenvector of ${\tilde{M}}_{t}$, both associated with the second largest eigenvalue since it captures the largest amount of variation in the system. As $K_{r}^{t}$ and $Q_{i}^{t}$ are relative metrics [26], the industry-based complexity of micro-regions (IndECI) and the Industry Complexity Index (ICI) are defined after applying the following standardization:

$$\begin{array}{c} {\text{IndECI}}_{r}^{t}=\frac{K_{r}^{t}-\left\langle K^{t}\right\rangle }{\text{std}(K^{t})} \end{array}$$ (8a)

$$\begin{array}{c} {\text{ICI}}_{i}^{t}=\frac{Q_{i}^{t}-\left\langle Q^{t}\right\rangle }{\text{std}(Q^{t})} \end{array}$$ (8b)

The intuition behind IndECI is similar: sophisticated regions are diverse and employ workers in high-sophistication sectors (ICI), which are prevalent in only a few diverse regions. In other words, an industrially complex region employs its workers, on average, in industries that only a few other regions are competitive.

### Related density and closeness to complex activities

We measure the density *ω* of an activity with revealed comparative advantages around activity *i* in the portfolio of activities of region *r* as

$$\begin{array}{c} \omega_{ri}=\frac{\sum_{i^{\prime }}M_{ri^{\prime }}\phi_{ii^{\prime }}}{\sum_{i^{\prime }}\phi_{ii^{\prime }}} \end{array}$$ (9)

where *ϕ*_*ii*′_ = min(*P*(RCA_*r*,*i*_ ≥ 1|RCA_*r*,*i*′_ ≥ 1), *P*(RCA_*r*,*i*′_ ≥ 1|RCA_*r*,*i*_ ≥ 1) measures the proximity between two activities and is measured as the minimum conditional probability that a region has revealed comparative advantage in both activities. The proximities form the backbone of the Product Space [59] and the Industry Space [25]. Relatedness is a well-known predictor of the ability of regions to develop new activities [35], implying that regions are more likely to enter activities that are related to their current capabilities.

In order to analyse the distance of each region’s economic structure to simple/complex new activities, we measure the Pearson correlation between the measured density (*ω*) of activities without revealing comparative advantage in regions and the complexity of such activities (PCI or ICI) of these potential new activities. In that sense, a negative correlation indicates that the diversification opportunities of regions are mainly simple activities, a positive correlation indicates that the closest diversification opportunities are mainly complex activities, a null correlation indicates that the region is equally close to simple and complex activities [60, 61].

In this measure of “closeness to complex activities,” it is important to mention that we consider only activities for which a region has not yet exhibited a revealed comparative advantage. Such choice has two main reasons: first, the focus of these measures is on the future development opportunities at different levels of economic development; secondly, measuring distance/closeness to existing activities (*M*_*ri*_ = 1) is redundant as such activities already make up a region’s portfolio and thus contribute already to the regional complexity.

## Results and discussion

**Fig 1:**
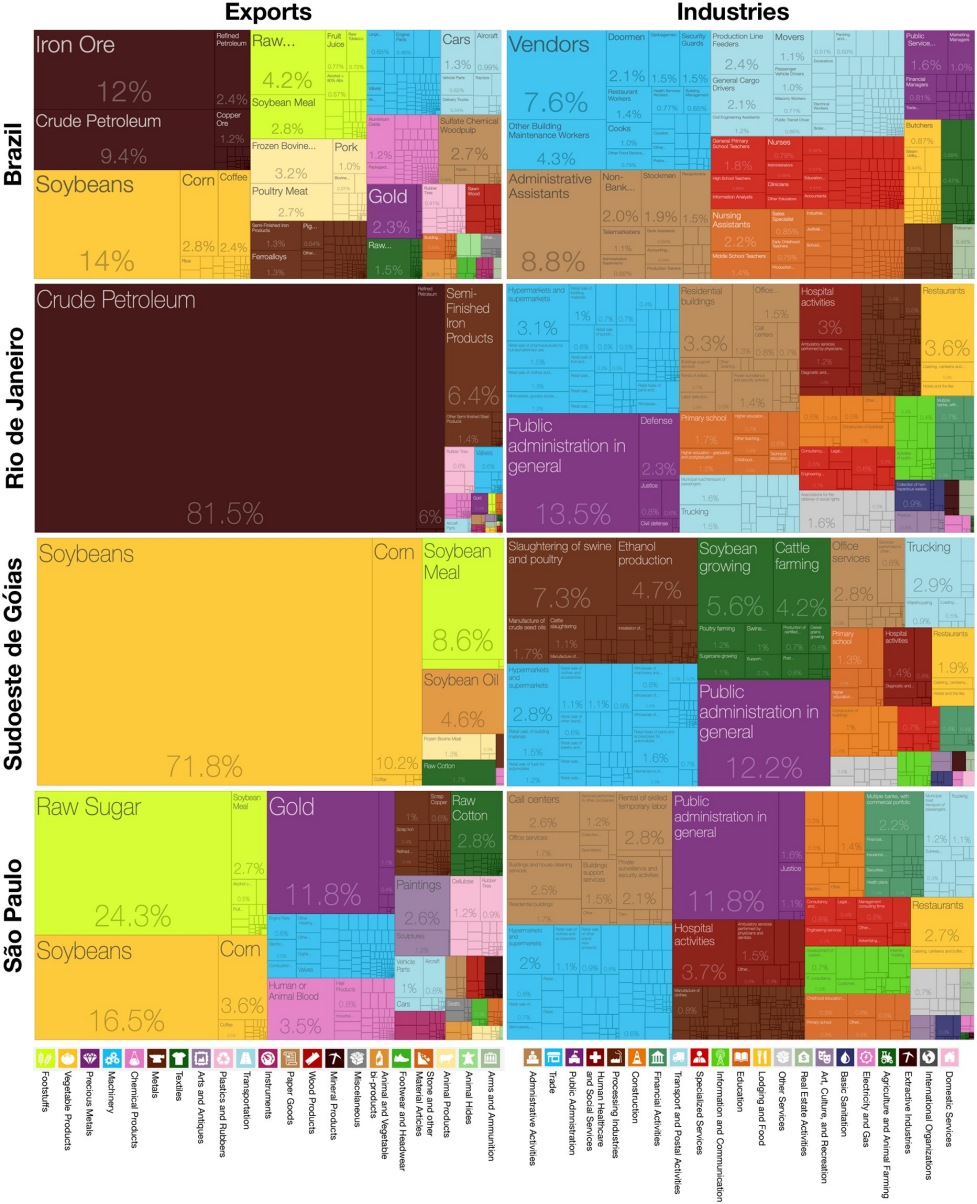
Export values in 2022 versus employment by industry data in 2021 for Brazil and the micro-regions Rio de Janeiro, the soybeans hub Sudoeste de Goias, and Sao Paulo. Brazil data at higher aggregation level ‘sections’ to better clarify product and industry types. Source, Dataviva.info [62, 63].

Brazil has an export structure strongly dependent on natural resources, such as soybeans, iron ore, or crude petroleum, see Fig 1. However, when looking at employment data, many people in Brazil work in different types of processing industries or service activities. For example, the almost exclusive emphasis on Rio de Janeiro’s exports of crude petroleum does, of course, not properly depict the actual labor markets with many people working in different types of service activities, including public administration, commerce, and tourism, but also research and higher education activities, banking, etc (Fig 1). São Paulo’s exports depict strengths in the export of cars, manufacturing, and chemical industries, but of course, a substantial share of these activities go to national clients. São Paulo is a hub for knowledge-based services (Fig 1). This raises the question of whether export complexity or industrial complexity captures productive capabilities and growth prospects in Brazil more adequately.

We start our analysis by comparing the industrial (ICI and IndECI) and export (PCI and ECI) complexity indicators and their correlation with economic growth. Table 1 shows several services featuring among the highest- and lowest-ranked products and industries regarding the respective complexity indicators. Agricultural and mining activities—such as simple commerce stores, herbs, and cocoa production—and simple services (*e.g*., retail) are ubiquitous across Brazilian micro-regions and, thus, score low in complexity. At the same time, several types of specialized financial services—such as investment banks or non-life insurance—and different manufacturing activities—such as vehicle parts, motors, or electronics—are less ubiquitous and thus achieve a high complexity score. The industrial and export complexity indices rank manufacturing activities as high complexity and most agricultural activities as low complexity.

**Table 1:** Activities (products and industries) in the bottom-5 and top-5 of complexity estimated from exports (top) and industry (bottom), and the region with the highest RCA on such activity.

**Product Complexity (Exports Data)**		
product	PCI	micro-region with highest RCA
1801: Cocoa (whole or broken), raw or roasted	-2.895	Tomé-Açu, PA (RCA = 34.1)
2609: Tin ores and concentrates	-2.874	Ariquemes, RO (RCA = 1523.0)
2602: Manganese ores & concentrates inc mangnfrs iron ores	-2.694	Chorozinho, CE (RCA = 2680.2)
2709: Crude oil from petroleum and bituminous minerals	-2.636	Baía da Ilha Grande, RJ (RCA = 18.2)
5201: Cotton, not carded or combed	-2.606	Tesouro, MT (RCA = 1270.3)
…	…	…
9010: Apparatus and equipment for photographic…	2.094	Barra do Piraí, RJ (RCA = 0.002)
8461: Machine-tools for planing, shaping, slotting,…	2.104	Itajubá, MG (RCA = 3.86)
3707: Chemical preparations for photographic uses…	2.217	Mogi das Cruzes, SP (RCA = 1.11)
8457: Machining centres, unit construction machines…	2.411	Sorocaba, SP (RCA = 3.50)
3705: Photographic plates and film, exposed and…	2.468	Jundiaí, SP (RCA = 2.99)
**Industry Complexity (Industry Data)**		
industry	ICI	micro-region with highest RCA
7227: Tin Ore Extraction	-2.310	Rio Preto da Eva, AM (RCA = 4408.5)
84116: General Public Administration	-2.105	Traipu, AL (RCA = 5.96)
7243: Precious Metal Ore Extraction	-2.056	S. Miguel do Araguaia, GO (RCA = 228.6)
3213: Aquaculture in salty and brackish water	-1.958	Litoral Sul, RN (RCA = 403.7)
47121: Retail trade of general merchandise …	-1.933	Oiapoque, AP (RCA = 8.82)
…	…	…
64328: Investment Banks	2.625	São Paulo, SP (RCA = 7.57)
64506: Capitalization Companies	2.899	Rio de Janeiro, RJ (RCA = 8.80)
30504: Manufacturing of Military Combat Vehicles	3.521	Campinas, SP (RCA = 49.54)
66118: Administration of Exchanges and…	3.671	Osasco, SP (RCA = 8.92)
64409: Commercial Leasing	3.671	Osasco, SP (RCA = 52.5)

**Fig 2:**
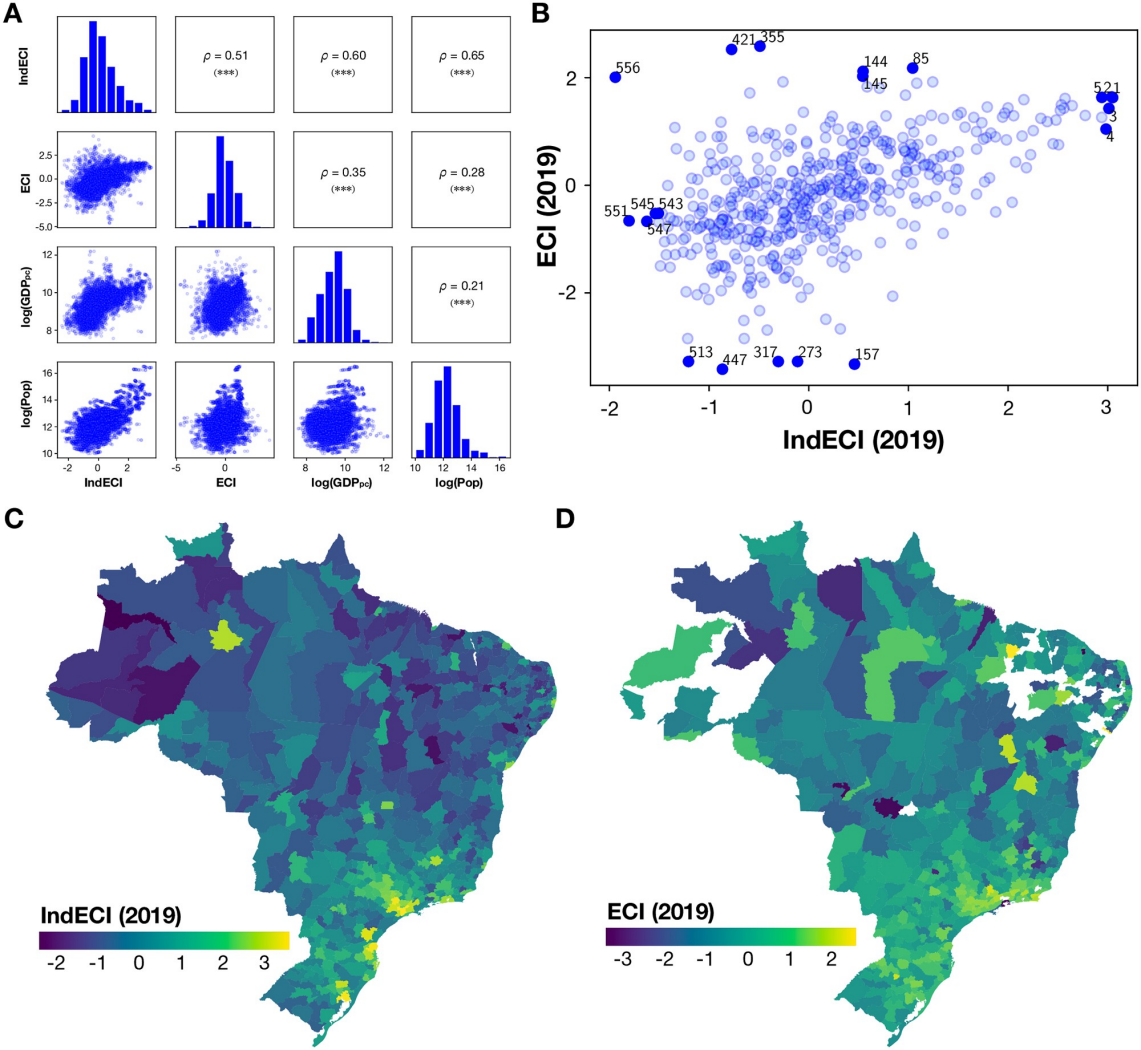
Panel A, Correlations between industrial complexity, export complexity, GDPpc, and population size of micro-regions. Panel B, Relationship between industry-based and export-based economic complexity index for 2019; the numbers correspond to the position in the IndECI ranking (Table 2). In darker blue, the top and bottom five micro-regions of both indicators. The numbers correspond to the position in the IndECI ranking. Panel C, the spatial distribution of industry-based economic complexity index for 2019. Panel D, Spatial distribution of export-based economic complexity index for 2019.

A more substantial difference between the industrial complexity index and the export complexity index rankings emerges when we look at their association with other variables, such as GDP and population. Only an intermediate correlation of *ρ* = 0.51 can be found between the industrial complexity and the export complexity index of the micro-regions (see Fig 2A for a general correlation matrix and Fig 2B for a more detailed scatter), and Table 2 shows that there is no match between the top and bottom five micro-regions in both rankings. It is noteworthy that more well-known places such as São Paulo, Campinas, or Porto Alegre appear among the top industrial complexity regions but are absent in the exports-based rank.

**Table 2:** Top and bottom regions based on their industry-based and export-based economic complexity index in 2019. The numbers in parentheses correspond to the position in the IndECI ranking. The list only contains micro-regions that have exports (and, therefore, an ECI value).

IndECI ranking		ECI ranking	
IndECI	Micro-region	ECI	Micro-region
3.0496	(1) Osasco	2.5853	(355) Codó
3.0405	(2) Guarulhos	2.5256	(421) Propriá
3.0120	(3) São Paulo	2.1786	(85) Santa Rita do Sapucaí
2.9832	(4) Porto Alegre	2.1187	(144) Não-Me-Toque
2.9411	(5) Campinas	2.0276	(145) Bacia de São João
…	…	…	…
-1.5087	(543) Caracaraí	-3.2764	(513) Tesouro
-1.5386	(545) Boca do Acre	-3.2764	(273) Alto Paraguai
-1.6240	(547) Bico do Papagaio	-3.2764	(317) Esperança
-1.8027	(551) Chapadas das Mangabeiras	-3.3258	(157) Baía da Ilha Grande
-1.9403	(556) Cotegipe	-3.4197	(447) Chorozinho

There is a higher correlation between industrial complexity and GDP per capita (*ρ* = 0.60) than between export complexity and GDP per capita (*ρ* = 0.51). A significantly stronger association can be observed between industrial complexity and population size (*ρ* = 0.65) than between export complexity and population size (*ρ* = 0.21).

Both regional economic complexity indices tend to be higher in Brazil’s Southern and Southeastern regions than in the Northern and North-Eastern regions, with the notable exception of several relatively complex larger cities in the latter regions. However, the spatial maps also illustrate that export complexity values are more dispersed than industrial complexity, which is more centered around metropolitan areas, such as São Paulo, Porto Alegre, Manaus, or Joinville, and more skewed towards the population denser coastal areas than the export complexity index (see Fig 2C and 2D).

This points to a problem of the export complexity index (ECI) as an indicator for knowledge intensity in a country with large levels of spatial heterogeneity and inequality like Brazil, in which very few regions (such as Santa Rita do Sapucaí or Não-Me-Toque) de facto produce and focus on high export complexity goods. But even export cluster regions such as Santa Rita do Sapucaí focus on “Glass mirrors” and have an export complexity index of 2.18, or Não-Me-Toque, focuses on the production of “Agricultural machinery for soil preparation or cultivation” and an ECI index of 2.12. For many regions in Brazil, it is enough to export intermediate to low-level export goods like pork and chicken meat or textile products to be considered relatively complex compared to regions that export none or even simpler products.

While this might make sense from a “first steps of industrialization” perspective, it is also slightly at odds with a modern understanding of a knowledge-based society in which high-skilled workers work in knowledge-based services activities in urban centers with a high density of higher education facilities, access to diverse ideas, and a differentiated demand structure [64–67].

**Fig 3:**
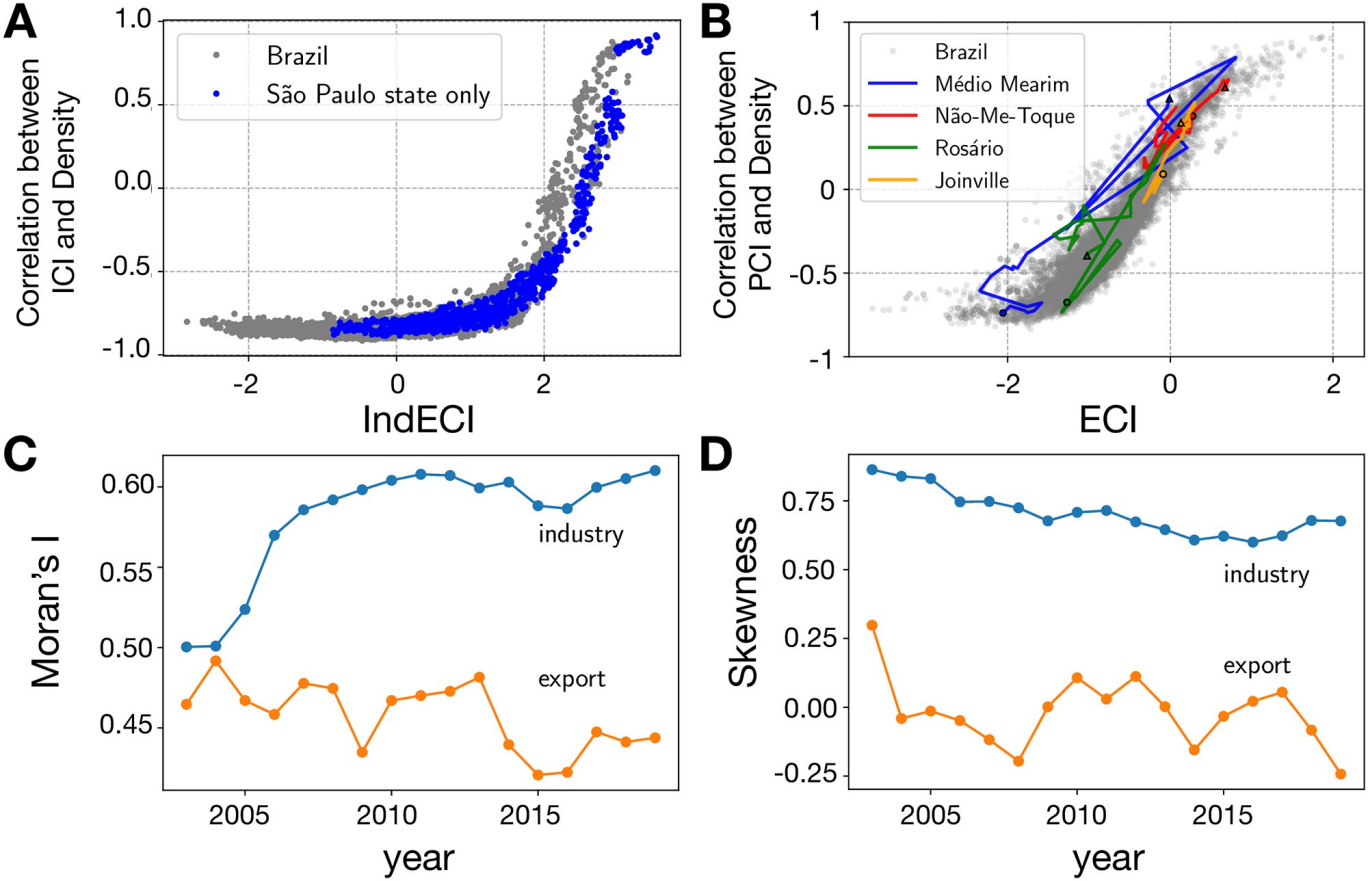
The S-curve of economic development for both industry-based (Panel A) and export-based (Panel B) economic complexity indexes depicting the closeness of each micro-region to complex activities given their complexity level. Panel C shows the time evolution of spatial autocorrelation (Moran’s I), and Panel D shows the spatial concentration (skewness) of both complexity metrics.

To further understand the distribution of industrial and export complexity, we plot in Fig 3A and 3B the regional complexity indices on the horizontal axis versus the closeness of the regions to new complex activities on the Y-axis. Previous work on this usually S-shaped association/curve of productive sophistication [20, 35, 55, 60, 68] showed that countries and regions tend to first diversify in the initial stages of development first into related, relatively simple activities, and only at intermediate to high levels of economic development move closer to complex activities. This implies a certain gravitation of regions with simple productive structures towards diversification into simple activities and of complex regions towards complex activities [5, 59].

We show a more linear association between the export complexity index (ECI) and the closeness to export products across micro-regions of Brazil than between industry complexity (IndECI) and closeness to complex activities. There is a very steep slope beyond a relatively high industrial complexity index. Only a few Brazilian micro-regions (mainly some urban centers) are close to the most complex industries. Moreover, it is noteworthy that industrial complexity evolution is more stable than export complexity evolution, and a significant variation in the growth rates of industrial versus export complexity between 2003 and 2019 can be observed. Regions like Joinville illustrate a certain correlation in the trend of both variables. However, in other cases (such as Médio Mearim) big jumps have been made in export complexity, but little progress has been made in industrial complexity. Initial levels of industrial complexity are different, more stable, and better predictors of future economic development trajectories.

Regarding the spatial distribution of complexity indices. Fig 3C shows the Moran’s I Index [69], which is computed as

$$\begin{array}{c} \text{Morans}’\;\text{I}=\frac{\left||R|\right|}{\sum_{r\in R}\left||N\left(r\right)|\right|}\frac{\sum_{r\in R}\sum_{r^{\prime }\in N\left(r\right)}\left({\text{ECI}}_{r^{\prime }}-\left\langle \text{ECI}\right\rangle \right)\left({\text{ECI}}_{r}-\left\langle \text{ECI}\right\rangle \right)}{\sum_{r\in R}{\left({\text{ECI}}_{r}-\left\langle \text{ECI}\right\rangle \right)}^{2}} \end{array}$$ (10)

where *R* is the set of 558 micro-regions and N(r) is the set of neighbor micro-regions of micro-region *r*. The values of Morans’ I range from -1 to +1 and quantify spatial autocorrelations. A negative spatial autocorrelation is associated with patterns like a chessboard (in which white squares surround all black squares and vice versa) and positive autocorrelation with regions neighboring other regions that are similar. A random arrangement would lead to a Moran’s I value close to 0.

Moreover, regarding the distribution of the complexity indexes, Fig 3D shows the right-skewness, which can be estimated as

$$\begin{array}{c} \text{skewness}=\frac{\left\langle {\left({\text{ECI}}_{r}-\left\langle \text{ECI}\right\rangle \right)}^{3}\right\rangle }{{\left\langle {\left({\text{ECI}}_{r}-\left\langle \text{ECI}\right\rangle \right)}^{2}\right\rangle }^{\frac{3}{2}}} \end{array}$$ (11)

Fig 3D shows that, during the analysis period, industrial complexity (IndECI) exhibits consistently higher levels of right-side skewness than export complexity (ECI), which indicated that IndECI is shifted towards higher values on the right side of the distribution. Additionally, as shown in Fig 3C, IndECI exhibits higher spatial autocorrelations, measured in terms of the Morans’ I index, than export complexity (ECI). This means that neighboring regions more often have similar values in terms of industry complexity than export complexity. However, in both cases, spatial spillover effects are likely to affect the economic behavior of the micro-regions. Notably, the spatial autocorrelations of regional export complexity have slightly reduced from 0.46 in 2003 to 0.44 in 2019, and the spatial autocorrelation of industry complexity has significantly gone up from 0.50 in 2003 to 0.61 in 2019.

### Regional complexity and economic growth

Next, we analyse the role of each economic complexity indicator in the economic growth prospects of each region. We quantify how much the economic complexity of a focal region and its neighboring regions impact the economic growth of the focal region. To that end, we define the average complexity of the neighbors of a focal region simply as:

$$\begin{array}{c} {\left\langle \text{ECI}\right\rangle }_{N(r)}^{t}=\frac{1}{\left||N(r)|\right|}\sum_{r^{\prime }\in N\left(r\right)}{\text{ECI}}_{r^{\prime }}^{t} \end{array}$$ (12)

We then follow with a regression analysis to examine the link between the economic growth of a focal region with its industry- and export-based economic complexity and the average complexity of their geographical neighbors. We also control the micro-regional real GDP per capita ($y_{r}^{t}$) and micro-regional population size ($N_{r}^{t}$). We define the continuous-time-equivalent 3-year growth rate as $g_{r}^{t}=\left(\text{log}y_{r}^{t+3}-\text{log}y_{r}^{t}\right)/3$. Results are robust considering 2- and 4-year regional growth, as shown in the S1 File.

In the end, we focus our analysis on the following model that regresses economic growth ($g_{r}^{t}$) against a set of relevant independent variables (described above), such that:

$$\begin{array}{c} \begin{array}{cc} g_{r}^{t}= & \beta_{0}+\beta_{1}{\text{IndECI}}_{r}^{t}+\beta_{2}{\left\langle \text{IndECI}\right\rangle }_{N(r)}^{t}+\beta_{3}{\text{ECI}}_{r}^{t}+\beta_{4}{\left\langle \text{ECI}\right\rangle }_{N(r)}^{t}+\\ & \beta_{5}\text{log}y_{r}^{t}+\beta_{6}\text{log}N_{r}^{t}+\rho g_{r}^{t-1}+\mu_{r}+\nu^{t}+\epsilon_{r}^{t} \end{array} \end{array}$$ (13)

where *μ*_*r*_ and *ν*^*t*^ are regional and year-fixed effects, and the residuals $\epsilon_{r}^{t}$ are assumed to be exogenous. Because we control these fixed effects, our model can capture the effect of different types of time-invariant characteristics of micro-regions and nationwide trends with year-fixed effects.

To detect the presence of autocorrelation, heteroscedasticity, multicollinearity, and endogeneity of the variables (Allano and Bond, Breusch-Pagan), we assessed the variance inflation factor (VIF) and performed a Durbin-Wu-Hausman tests. Based on these tests, it was verified that the dataset used presents the following characteristics that must be treated so that the estimates are consistent: a) the dependent variable is dynamic, in the sense that it depends on its past values; b) the independent variables are not strictly exogenous; c) there are fixed effects in observational units (micro-regions) and time periods, which must be controlled; d) multicollinearity between ECI and IndECI.

Hence, considering regional and temporal fixed effects, a dynamic Arellano–Bover/Blundell–Bond panel (Rodman, 2009) was used. This estimator is appropriate for the data set used, given that we have: a) a greater number of units than years; b) we consider a linear functional relation; c) there is an autoregressive coefficient; d) the independent variables are not strictly exogenous; e) there is a regional and temporal fixed effect. After estimation, both a Sargan and Sargan-Hansen test reject the null hypothesis of over-identification of the instruments and the validity of the instruments used. The Arellano-Bond test allows for rejecting the null hypothesis of serial correlation Instruments were used to orthogonal deviations equation and for the levels equation. General List of instruments used (in different models, different instruments from this list): FOD.log*N*, FOD.*logy*, L(2/3).*IndECI* collapsed, L(2/3).L.*IndECI* collapsed, L(2/3).*g* collapsed, DL.*IndECI* collapsed, DL.L.*IndECI* collapsed, DL.*g* collapsed, L(2/3).(〈*IndECI*〉_*N*_ L.〈*IndECI*〉_*N*_) collapsed, L(2/3).(*ECI* L.*ECI*) collapsed, DL.(*ECI* L.*ECI*) collapsed, L(2/3)(〈*ECI*〉_*N*_ L.〈*ECI*〉_*N*_) collapsed, DL.(〈*ECI*〉_*N*_ L.〈*ECI*〉_*N*_) collapsed, L(2/3).*g* collapsed. Given that the lags are collapsed, they are combined to reduce the total number of instruments, which helps avoid excessive instrument proliferation and related issues. Table 3 shows the results of the regression model. The first two columns show the model results with only the control variables. In the case of Column 1, the three-year lagged average growth rate is positive and significant, as expected. When we include GDP per capita and population size in Column 2, the result of the lagged dependent variable remains significant and with the same sign. Including these control variables is justified to absorb the scale of the region and the richness.

**Table 3:** Results of the regression model showing estimates of the average marginal effect of the local economic complexity index with neighbors’ terms.

GDP growth, *g*^*t*^								
	(1)	(2)	(3)	(4)	(5)	(6)	(7)	(8)
IndECI			0.0987*** (0.0198)		0.0427*** (0.0147)			
〈IndECI〉_*N*_				0.1526*** (0.0173)	0.1056*** (0.0147)			
ECI						0.0041 (0.0092)		0.0044 (0.0071)
〈ECI〉_*N*_							0.1330*** (0.0164)	0.1199*** (0.0157)
log *y*		-0.4789*** (0.0501)	-0.4182*** (0.0369)	-0.3771*** (0.0322)	-0.3254*** (0.0266)	-0.4406*** (0.0434)	-0.3593*** (0.0337)	-0.3357*** (0.0313)
log *N*		-0.2672*** (0.0702)	-0.2140*** (0.5059)	-0.2025*** (0.0504)	-0.1299*** (0.0339)	-0.2271*** (0.0598)	-0.2234*** (0.0565)	-0.2020*** (0.0502)
*g* ^*t*−1^	0.6393*** (0.0320)	0.3663*** (0.0450)	0.4113*** (0.0365)	0.4062*** (0.0333)	0.4447*** (0.0301)	0.3947*** (0.0387)	0.4151*** (0.0319)	0.4312*** (0.0302)
Constant	0.0931*** (0.0063)	7.7413*** (1.0896)	6.5252*** (0.7819)	5.9851*** (0.739)	4.6335*** (0.5153)	6.9039 (0.9141)	6.4831*** (0.8511)	5.9746*** (0.7619)
Fixed Effects								
Micro-region	Yes	Yes	Yes	Yes	Yes	Yes	Yes	Yes
Year	Yes	Yes	Yes	Yes	Yes	Yes	Yes	Yes
AB test for AR(1)	0.98	1.32	-3.33	-4.31	-2.39	-1.86	-3.12	1.53
AB test for AR(2)	-0.87	-1.57	0.93	0.55	0.78	0.526	1.73	-1.1
Sargan Test	44.02	10.15	2.58	3.75	1.19	8.79	34.56	2.69
Observations	5773	5773	5773	5773	5773	5773	5773	5773

The complexity variable IndECI Is included in column 3. As expected, the coefficient is positive (and significant), which indicates that the region’s growth rate is positively related to a higher industrial complexity indicator. On the other hand, in Column 4, it is noticed that there are neighborhood spillovers, given that a higher indicator of 〈IndECI〉_*N*_ of the neighbors of each micro-region is also positively related to the average growth rate. In Column 5, the two variables IndECI and 〈IndECI〉_*N*_ are combined, and the results remain significant. However, we have a decrease in the complexity coefficient of the region, which is lower than the complexity indicator of the neighbors. Columns 6, 7, and 8 present the results when considering the variables related to exports from the micro-region and close neighbors. Only the second of these variables was significant and with the expected sign.

Thus, an increase in the export complexity of neighboring regions is significantly associated with an increase in the economic growth prospects of the target region. Still, an increase in export complexity within the region is insufficient to raise GDP per capita significantly (at least within the analysed time period).

**Fig 4:**
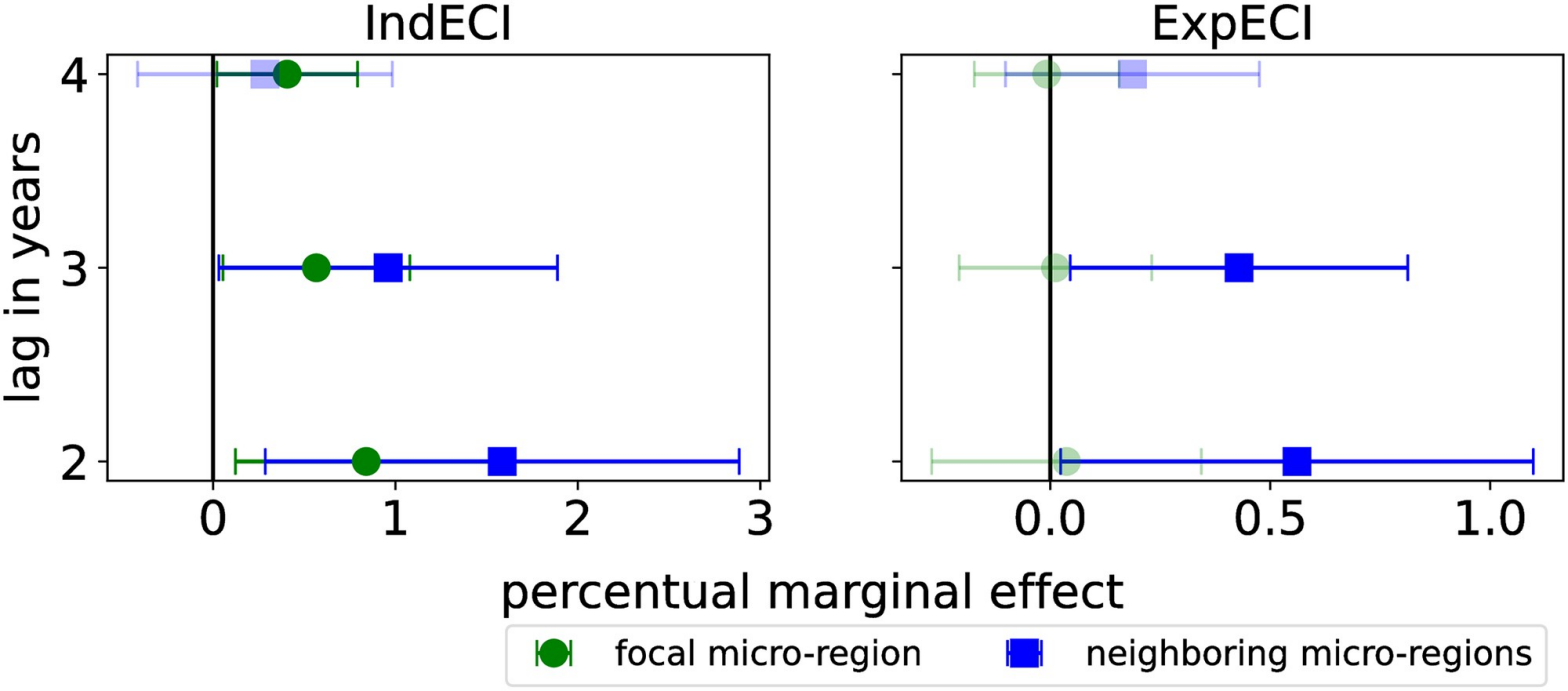
The marginal effect in different time lag growth.

To test the robustness of the thesis of this work, we considered different time intervals to measure the growth of micro-regions. As Fig 4 shows, we see that the results are quite general: The industrial complexity of a location and its neighborhood are important for growth, while only the complexity of the neighborhood’s exports matters for a location’s growth. The regression table for all these results can be found in the S1 File.

These results imply that an economic diversification strategy that only considering exports may not be sufficient to use local knowledge, smart diversification, and growth potential in a large developing/emerging economy such as Brazil. Instead of identifying with complete industrial data, for instance, significant opportunities for regions to move into knowledge-based services or manufacturing activities for the large Brazilian market, export data can lead to identifying relatively simple, natural resource-exploiting goods [70]. This could further perpetuate the traditional problems of external dependency and focus on comparative advantages in natural resources, agricultural, and mining activities instead of structural transformation into more knowledge-based and value-added activities [43, 44, 68].

## Conclusions

Initial approaches from economic complexity research have tended to focus on export structures as indicators of productive structures [1, 58], as it best fits available data and it is in line with literature highlighting the problem of core-periphery structures in trade and the problem of external dependence of countries [44, 45, 68, 71]. The application of complexity indicators to regions of developed economies have tended to use patent data as a proxy for the technology frontier [18, 55].

In this article, we show that industrial complexity is more adequate at capturing the knowledge base and predicting economic growth at the micro-regional level in a large emerging economy, such as Brazil, than export data. Export data does not capture significant strengths of regions in services as well as manufacturing activities destined for the large domestic 1market. Indeed, a large share of the workforce does not work in export-sectors and thus export data strongly underestimates the actual knowledge and skills of the workforce.

Our results show a stronger spatial concentration and skewness of industrial complexity values than export complexity values in Brazil. Only a few urban hubs have managed to achieve comparative advantages in the most complex services and specialized manufacturing activities, while low to intermediately complex agro-industrial exports are already sufficient to achieve relatively high economic complexity values in the within-country export complexity comparisons. Industrial complexity is a strong predictor of regional economic growth, however, the export complexity of a region is not, at least in the case of Brazil.

These results invite a rethinking of complexity as a policy tool in the context of regional development policies. Indeed, complexity-based indicators are multidimensional and highly dependent on the choice of underlying proxy data. In effect, they capture different dimensions of productive systems and knowledge bases, which arguably should be evaluated as separate, complementary, and/or interrelated challenges in future work. Simply put, increasing the export complexity of a region does not directly entail improving the region’s industry complexity, or vice versa. In the case of countries, such as Brazil, a mere emphasis on exports is not sufficient to lift all regional boats, and endogenous smart diversification opportunities are not identified. This does not mean that an increase in more knowledge- and technology-intensive exports should not be part of smart diversification strategies of Brazil. Yet it is not sufficient to ensure economic growth across the heterogeneous regions of large emerging economies, such as Brazil.

In conclusion, our study shows that considering industrial complexity, which includes all employment sectors, offers a more accurate foundation for designing industrial policies in emerging economies like Brazil. This approach captures the entire range of workforce skills and productive capacities, including non-tradables and sophisticated sectors, such as software development and graphic design. By focusing on industrial complexity, policymakers can better understand and support a region’s diverse productive landscape, promoting broader economic growth and resilience.

This study shed some light on the complicated matter of finding the right economic complexity dimension to promote economic growth across the regions of a large emerging economy. Future research arguably needs to benchmark results from countries at different stages of economic development and with different domestic market sizes. The study focused on the Brazilian context. Therefore, the findings may not be directly applicable to other emerging economies with different characteristics and economic structures. For example, smaller economies with underdeveloped domestic markets may require a larger scale to develop industries, making them more dependent on external markets to increase the complexity of their productive structure. A second limitation of the paper is that the study uses formal labor market data, which might exclude a significant part of the Brazilian economy operating in informality, especially in less developed regions.

## Supporting information

S1 File Contains additional results of the regression model for 2-year, 3-year, and 4-year growth showing estimates of the average marginal effect of the local economic complexity index. (PDF)

## References

[1] Hidalgo CA, Hausmann R. The building blocks of economic complexity. Proceedings of the national academy of sciences. 2009;106(26):10570–10575. doi: 10.1073/pnas.0900943106 19549871 PMC2705545

[2] Morrison G, Buldyrev SV, Imbruno M, Doria Arrieta OA, Rungi A, Riccaboni M, et al. On economic complexity and the fitness of nations. Scientific Reports. 2017;7(1):15332. doi: 10.1038/s41598-017-14603-6 29127304 PMC5681668

[3] Sbardella A, Zaccaria A, Pietronero L, Scaramozzino P. Behind the Italian regional divide: An economic fitness and complexity perspective. 2021;.

[4] Sciarra C, Chiarotti G, Ridolfi L, Laio F. Reconciling contrasting views on economic complexity. Nature communications. 2020;11(1):3352. doi: 10.1038/s41467-020-16992-1 32620815 PMC7335174

[5] Hausmann R, Hidalgo C. Country diversification, product ubiquity, and economic divergence. 2010;.

[6] Hartmann D, Guevara MR, Jara-Figueroa C, Aristarán M, Hidalgo CA. Linking economic complexity, institutions, and income inequality. World Development. 2017;93:75–93. doi: 10.1016/j.worlddev.2016.12.020

[7] Doğan B, Chu LK, Ghosh S, Truong HHD, Balsalobre-Lorente D. How environmental taxes and carbon emissions are related in the G7 economies? Renewable Energy. 2022;187:645–656. doi: 10.1016/j.renene.2022.01.077

[8] Ghosh S, Balsalobre-Lorente D, Doğan B, Paiano A, Talbi B. Modelling an empirical framework of the implications of tourism and economic complexity on environmental sustainability in G7 economies. Journal of Cleaner Production. 2022;376:134281. doi: 10.1016/j.jclepro.2022.134281

[9] Li Z, Doğan B, Ghosh S, Chen WM, Lorente DB. Economic complexity, natural resources and economic progress in the era of sustainable development: Findings in the context of resource deployment challenges. Resources Policy. 2024;88:104504. doi: 10.1016/j.resourpol.2023.104504

[10] Doğan B, Balsalobre-Lorente D, Nasir MA. European commitment to COP21 and the role of energy consumption, FDI, trade and economic complexity in sustaining economic growth. Journal of environmental Management. 2020;273:111146. 32771851 10.1016/j.jenvman.2020.111146

[11] Doğan B, Driha OM, Balsalobre Lorente D, Shahzad U. The mitigating effects of economic complexity and renewable energy on carbon emissions in developed countries. Sustainable Development. 2021;29(1):1–12. doi: 10.1002/sd.2125

[12] Albeaik S, Kaltenberg M, Alsaleh M, Hidalgo CA. Improving the economic complexity index. arXiv preprint arXiv:170705826. 2017;.

[13] Hausmann R, Hidalgo CA, Bustos S, Coscia M, Simoes A. The atlas of economic complexity: Mapping paths to prosperity. Mit Press; 2014.

[14] Hausmann R, Rodrik D. Economic development as self-discovery. Journal of development Economics. 2003;72(2):603–633. doi: 10.1016/S0304-3878(03)00124-X

[15] Stojkoski V, Utkovski Z, Kocarev L. The impact of services on economic complexity: Service sophistication as route for economic growth. PloS one. 2016;11(8):e0161633. doi: 10.1371/journal.pone.0161633 27560133 PMC4999235

[16] Freitas EE, et al. Indústrias relacionadas, complexidade econômica e diversificação regional: uma aplicação para microrregiões brasileiras. 2019;.

[17] Balland PA, Rigby D. The geography of complex knowledge. Economic geography. 2017;93(1):1–23. doi: 10.1080/00130095.2016.1205947

[18] Balland PA, Boschma R, Crespo J, Rigby DL. Smart specialization policy in the European Union: relatedness, knowledge complexity and regional diversification. Regional studies. 2018;.

[19] Petralia S, Balland PA, Morrison A. Climbing the ladder of technological development. Research Policy. 2017;46(5):956–969. doi: 10.1016/j.respol.2017.03.012

[20] Pinheiro FL, Balland PA, Boschma R, Hartmann D. The dark side of the geography of innovation: relatedness, complexity and regional inequality in Europe. Regional Studies. 2022; p. 1–16. doi: 10.1080/00343404.2022.2106362

[21] Guevara MR, Hartmann D, Aristarán M, Mendoza M, Hidalgo CA. The research space: using career paths to predict the evolution of the research output of individuals, institutions, and nations. Scientometrics. 2016;109:1695–1709. doi: 10.1007/s11192-016-2125-9

[22] Pugliese E, Cimini G, Patelli A, Zaccaria A, Pietronero L, Gabrielli A. Unfolding the innovation system for the development of countries: coevolution of Science, Technology and Production. Scientific reports. 2019;9(1):16440. doi: 10.1038/s41598-019-52767-5 31712700 PMC6848202

[23] Stojkoski V, Koch P, Hidalgo CA. Multidimensional economic complexity and inclusive green growth. Communications Earth & Environment. 2023;4(1):130. doi: 10.1038/s43247-023-00770-0

[24] Lo Turco A, Maggioni D. The knowledge and skill content of production complexity. Research Policy. 2022; 51(2).

[25] Gao J, Jun B, Pentland Aâ, Zhou T, Hidalgo CA. Spillovers across industries and regions in China’s regional economic diversification. Regional Studies. 2021;55(7):1311–1326. doi: 10.1080/00343404.2021.1883191

[26] Fritz BS, Manduca RA. The economic complexity of US metropolitan areas. Regional Studies. 2021;55(7):1299–1310. doi: 10.1080/00343404.2021.1884215

[27] Chávez JC, Mosqueda MT, Gómez-Zaldívar M. Economic complexity and regional growth performance: Evidence from the Mexican Economy. Review of Regional Studies. 2017;47(2):201–219.

[28] Freitas EE, Romero JP, Britto G, de Queiroz Stein A, Torres R. Dataviva: espaço de atividades e indicadores regionais de complexidade econômica. Cedeplar, Universidade Federal de Minas Gerais; 2023.

[29] Koch P, Schwarzbauer W. Yet another space: Why the Industry Space adds value to the understanding of structural change and economic development. Structural Change and Economic Dynamics. 2021;59:198–213. doi: 10.1016/j.strueco.2021.08.019

[30] Klinger B, Lederman D. Diversification, innovation, and imitation inside the global technological frontier. World Bank policy research working paper. 2006; (3872).

[31] Operti FG, Pugliese E, Andrade JS Jr, Pietronero L, Gabrielli A. Dynamics in the Fitness-Income plane: Brazilian states vs World countries. PloS one. 2018;13(6):e0197616. doi: 10.1371/journal.pone.0197616 29874265 PMC5991372

[32] Britto G, Romero JP, Freitas E, Coelho C. The great divide: economic complexity and development paths in Brazil and the Republic of Korea. Cepal Review. 2019;. doi: 10.18356/d06fff2f-en

[33] Ferraz D, Moralles HF, Campoli JS, Oliveira FCRd, Rebelatto DAdN. Economic complexity and human development: DEA performance measurement in Asia and Latin America. Gestão & Produção. 2018;25:839–853.

[34] Bandeira Morais M, Swart J, Jordaan JA. Economic complexity and inequality: Does regional productive structure affect income inequality in Brazilian states? Sustainability. 2021;13(2):1006. doi: 10.3390/su13021006

[35] Hartmann D, Zagato L, Gala P, Pinheiro FL. Why did some countries catch-up, while others got stuck in the middle? Stages of productive sophistication and smart industrial policies. Structural Change and Economic Dynamics. 2021;58:1–13. doi: 10.1016/j.strueco.2021.04.007

[36] Zhu S, Yu C, He C. Export structures, income inequality and urban-rural divide in China. Applied Geography. 2020;115:102150. doi: 10.1016/j.apgeog.2020.102150

[37] de Carvalho DE, de Queiroz Stein A, Queiroz AR, Romero JP. Complexidade econômica e crescimento do PIB per capita: uma análise de diferenças-em-diferenças para os municípios brasileiros. 2022;.

[38] Queiroz AR, Romero JP, Freitas E. Economic complexity and employment in Brazilian states. CEPAL Review. 2023;.

[39] Romero J, Freitas E, Silveira F, Britto G, Cimini F, Jayme G. Economic complexity and regional economic development: evidence from Brazil. In: EAEPE, Online Proceedings; 2021. p. 1–22.

[40] Arend M, Fonseca PCD. Brasil (1955-2005): 25 anos de catching up, 25 anos de falling behind. Brazilian Journal of Political Economy. 2012;32:33–54. doi: 10.1590/S0101-31572012000100003

[41] Arend M, Fagotti VZ, Guerrero GA, Fonseca PCD, da Silva Bichara J. Development strategies and path dependence: Institutional elements for making sense of Brazil’s falling behind and South Korea’s forging ahead. PSL Quarterly Review. 2023;76(305):155–180.

[42] Torezani TA. Produtividade da indústria brasileira: decomposição do crescimento e padrões de concentração em uma abordagem desagregada, 1996-2016. Revista Brasileira de Inovação. 2020;19:e0200029. doi: 10.20396/rbi.v19i0.8658100

[43] Furtado C. Formação econômica do Brasil. Companhia das Letras; 2020.

[44] Santos TD. The structure of dependence. The american economic review. 1970;60(2):231–236.

[45] Prebisch R. Commercial policy in the underdeveloped countries. the American economic review. 1959;49(2):251–273.

[46] Bielschowsky R. Sesenta años de la CEPAL: estructuralismo y neoestructuralismo. 2009;.

[47] Stojkoski V, Koch P, Coll E, Hidalgo CA. Estimating digital product trade through corporate revenue data. Nature Communications. 2024;15(1):5262. doi: 10.1038/s41467-024-49141-z 38897987 PMC11186826

[48] Baines TS, Lightfoot HW, Benedettini O, Kay JM. The servitization of manufacturing: A review of literature and reflection on future challenges. Journal of manufacturing technology management. 2009;20(5):547–567. doi: 10.1108/17410380910960984

[49] The Growth Lab at Harvard University. “Growth Projections and Complexity Rankings, V2” [Dataset];. https://dataverse.harvard.edu/.

[50] COMEX. Ministério do Desenvolvimento, Indústria e Comércio Exterior. 2015;.

[51] IBGE. Divisão do Brasil em mesorregiões e microrregiões geográficas; 2022. https://biblioteca.ibge.gov.br/visualizacao/livros/liv2269_1.pdf.

[52] WIPO. intellectual property activity around the world; 2023. https://www.wipo.int/en/ipfactsandfigures/patents.

[53] IBGE. Produto interno bruto dos municípios.; 2022. https://www.ibge.gov.br/estatisticas/economicas/contas-nacionais/9088-produto-interno-bruto-dosmunicipios.html.

[54] IBGE. Preços e custos.; 2022. https://www.ibge.gov.br/estatisticas/economicas/precos-e-custos.html.

[55] Pinheiro FL, Hartmann D, Boschma R, Hidalgo CA. The time and frequency of unrelated diversification. Research Policy. 2022;51(8):104323. doi: 10.1016/j.respol.2021.104323

[56] Hoen AR, Oosterhaven J. On the measurement of comparative advantage. The Annals of Regional Science. 2006;40:677–691. doi: 10.1007/s00168-006-0076-4

[57] Simoes AJG, Hidalgo CA. The economic complexity observatory: An analytical tool for understanding the dynamics of economic development. In: Workshops at the twenty-fifth AAAI conference on artificial intelligence; 2011.

[58] Hidalgo CA. Economic complexity theory and applications. Nature Reviews Physics. 2021;3(2):92–113. doi: 10.1038/s42254-020-00275-1

[59] Hidalgo CA, Klinger B, Barabási AL, Hausmann R. The product space conditions the development of nations. Science. 2007;317(5837):482–487. doi: 10.1126/science.1144581 17656717

[60] Pinheiro FL, Alshamsi A, Hartmann D, Boschma R, Hidalgo C, et al. Shooting low or high: do countries benefit from entering unrelated activities? Papers in Evolutionary Economic Geography. 2018;18(07).

[61] Hartmann D, Bezerra M, Pinheiro FL. Identifying smart strategies for economic diversification and inclusive growth in developing economies. The case of Paraguay. The Case of Paraguay (March 5, 2019). 2019;.

[62] Cedeplar—UFMJ. Data Viva;. https://www.dataviva.info.

[63] Hidalgo C. Big data visualization engines for understanding the development of countries, social networks, culture and cities. In: Proceedings of the 25th ACM conference on Hypertext and social media; 2014. p. 3–3.

[64] Jacobs J. The death and life of great American cities. Vintage; 2016.

[65] Florida R. The rise of the creative class. vol. 9. Basic books New York; 2002.

[66] Glaeser EL, Kallal HD, Scheinkman JA, Shleifer A. Growth in cities. Journal of political economy. 1992;100(6):1126–1152. doi: 10.1086/261856

[67] Giovanini A, Pereira WM, Almeida HJF. Productive diversity and economic growth: some evidence for Brazilian municipalities. Nova Economia. 2023;32:687–717. doi: 10.1590/0103-6351/6870

[68] Hartmann D, Bezerra M, Lodolo B, Pinheiro FL. International trade, development traps, and the core-periphery structure of income inequality. EconomiA. 2020;21(2):255–278. doi: 10.1016/j.econ.2019.09.001

[69] Moran PA. Notes on continuous stochastic phenomena. Biometrika. 1950;37(1/2):17–23. doi: 10.1093/biomet/37.1-2.17 15420245

[70] Hartmann D, Cardoso BHF, Arend M, Catela E. Complexidade setorial e estratégias de diversificação inteligente a nivel microrregional: o caso de Santa Catarina. 2023;.

[71] Gala P, Camargo J, Freitas E. The Economic Commission for Latin America and the Caribbean (ECLAC) was right: scale-free complex networks and core-periphery patterns in world trade. Cambridge Journal of Economics. 2018;42(3):633–651. doi: 10.1093/cje/bex057

