# Supplementary material for: Did industrial and export complexity drive regional economic growth in Brazil?
Source: PLoS One. 2024 Dec 5;19(12):e0313945. doi: 10.1371/journal.pone.0313945 (PMC11620402; doi:10.1371/journal.pone.0313945)
Supplement: S1 File — (PDF) [file pone.0313945.s001.pdf]

Supporting Information

In our study, we have demonstrated the impact of the Economic Complexity Index on the economic growth rate of the GDP in Brazilian micro-regions. Specifically, we examined the effects of this index at both the local and neighborhood levels, utilizing a 3-year lag to observe its impact. To ensure the robustness of our findings, we conducted additional analyses involving two distinct time frames, considering growth in 2 and 4-year lags. These methodological variations allowed us to provide a more comprehensive and nuanced understanding of the relationship between economic complexity and the economic development of Brazilian micro-regions.

First, we define the continuous-time-equivalent τ-year growth rate as $g_{r,\tau}^{(t)} = (\log y_r^{(t+\tau)} - \log y_r^{(t)}) / \tau$. Then, we make the OLS regressions:

$$g_{r,\tau}^{(t)} = \beta_0 + \beta_1 \mathrm{IndECI}_r^{(t)} + \beta_2 \langle \mathrm{IndECI} \rangle_{N(r)}^{(t)} + \beta_3 \mathrm{ECI}_r^{(t)} + \beta_4 \langle \mathrm{ECI} \rangle_{N(r)}^{(t)} + \beta_5 \log y_r^{(t)} + \beta_6 \log N_r^{(t)} + \mu_r + \upsilon^{(t)} + \varepsilon_r^{(t)}$$

Tables S1, S2, and S3 display the regression outcomes, with a focus on growth over 2, 3, and 4-year lags, respectively.

Table S1. Results of the regression model for 2-year growth showing estimates of the average marginal effect of the local economic complexity index.

	2-year growth					
	(1)	(2)	(3)	(5)	(6)	(7)
IndECI	0.0109***		0.0084**			
	(0.0035)		(0.0037)			
$\langle \mathrm{IndECI} \rangle_N$		0.0201***	0.0159**			
		(0.0064)	(0.0066)			
ECI				0.0005		
				(0.0016)		(0.0016)
$\langle \mathrm{ECI} \rangle_N$						
					(0.0027)	(0.0027)
$\log y$	-0.3051***	-0.3042***	-0.3044***	-0.3050***	-0.3047***	-0.3046***
	(0.0067)	(0.0067)	(0.0067)	(0.0067)	(0.0067)	(0.0067)
$\log \mathrm{N}$	-0.1496***	-0.1441***	-0.1427***	-0.1542***	-0.1523***	-0.1522***
	(0.0190)	(0.0192)	(0.0192)	(0.0189)	(0.0190)	(0.0190)
Constant	4.8363***	4.7711***	4.7480***	4.9010***	4.8764***	4.8758***
	(0.2667)	(0.2690)	(0.2691)	(0.2661)	(0.2663)	(0.2663)
micro-region f.e.	✓	✓	✓	✓	✓	✓
year f.e.	✓	✓	✓	✓	✓	✓
R^2	0.4915	0.4916	0.4921	0.4906	0.4910	0.4910
Adjusted R^2	0.4393	0.4393	0.4398	0.4383	0.4387	0.4386
Observations	5773	5773	5773	5773	5773	5773

Table S2. Results of the regression model for 3-year growth showing estimates of the average marginal effect of the local economic complexity index.

	3-year growth					
	(1)	(2)	(3)	(5)	(6)	(7)
IndECI	0.0071***	0.0057**				
	(0.0025)		(0.0026)			
$\langle \text{IndECI} \rangle_N$	0.0125***	0.0096**				
		(0.0045)	(0.0047)			
ECI			0.0002		0.0001	
				(0.0011)		(0.0011)
$\langle \text{ECI} \rangle_N$			0.0043**	0.0043**		
					(0.0020)	(0.0020)
$\log y$	-0.2383***	-0.2378***	-0.2379***	-0.2383***	-0.2380***	-0.2380***
	(0.0048)	(0.0048)	(0.0048)	(0.0048)	(0.0048)	(0.0048)
$\log \text{N}$	-0.1121***	-0.1089***	-0.1079***	-0.1151***	-0.1136***	-0.1136***
	(0.0136)	(0.0137)	(0.0137)	(0.0135)	(0.0135)	(0.0135)
Constant	3.7334***	3.6955***	3.6799***	3.7763***	3.7571***	3.7570***
	(0.1905)	(0.1922)	(0.1923)	(0.1901)	(0.1902)	(0.1902)
micro-region f.e.	✓	✓	✓	✓	✓	✓
year f.e.	✓	✓	✓	✓	✓	✓
R^2	0.5822	0.5821	0.5825	0.5815	0.5819	0.5819
Adjusted R^2	0.5392	0.5392	0.5395	0.5385	0.5390	0.5389
Observations	5773	5773	5773	5773	5773	5773

Table S3. Results of the regression model for 4-year growth showing estimates of the average marginal effect of the local economic complexity index with neighbors' terms.

	4-year growth					
	(1)	(2)	(3)	(5)	(6)	(7)
IndECI	0.0045**		0.0041**			
	(0.0019)		(0.0020)			
$\langle \text{IndECI} \rangle_N$		0.0049	0.0028			
		(0.0034)	(0.0036)			
ECI				-0.0000		
				(0.0008)		(0.0008)
$\langle \text{ECI} \rangle_N$						
					(0.0015)	(0.0015)
$\log y$	-0.2035***	-0.2033***	-0.2034***	-0.2035***	-0.2034***	-0.2034***
	(0.0036)	(0.0036)	(0.0036)	(0.0036)	(0.0036)	(0.0036)
$\log \text{N}$	-0.0987***	-0.0981***	-0.0974***	-0.1006***	-0.0999***	-0.0999***
	(0.0102)	(0.0103)	(0.0103)	(0.0102)	(0.0102)	(0.0102)
Constant	3.2468***	3.2421***	3.2309***	3.2741***	3.2656***	3.2657***
	(0.1433)	(0.1446)	(0.1447)	(0.1429)	(0.1430)	(0.1431)
micro-region f.e.	✓	✓	✓	✓	✓	✓
year f.e.	✓	✓	✓	✓	✓	✓
R^2	0.6759	0.6756	0.6759	0.6755	0.6756	0.6756
Adjusted R^2	0.6425	0.6423	0.6425	0.6421	0.6423	0.6422
Observations	5773	5773	5773	5773	5773	5773
